# Luteinizing Hormone Involvement in Aging Female Cognition: Not All Is Estrogen Loss

**DOI:** 10.3389/fendo.2018.00544

**Published:** 2018-09-24

**Authors:** Sabina Bhatta, Jeffrey A. Blair, Gemma Casadesus

**Affiliations:** ^1^School of Biomedical Sciences, Kent State University, Kent, OH, United States; ^2^Department of Biological Sciences, Kent State University, Kent, OH, United States

**Keywords:** luteinizing hormone, luteinizing hormone receptor, cognition, menopause, estrogen, ovariectomy, inverse relationship

## Abstract

Pervasive age-related dysfunction in hypothalamic-pituitary-gonadal (HPG) axis is associated with cognitive impairments in aging as well as pathogenesis of age-related neurodegenerative diseases such as the Alzheimer's disease (AD). As a major regulator of the HPG axis, the steroid hormone estrogen has been widely studied for its role in regulation of memory. Although estrogen modulates both cognition as well as cognition associated morphological components in a healthy state, the benefits of estrogen replacement therapy on cognition and disease seem to diminish with advancing age. Emerging data suggests an important role for luteinizing hormone (LH) in CNS function, which is another component of the HPG axis that becomes dysregulated during aging, particularly in menopause. The goal of this review is to highlight the current existing literature on LH and provide new insights on possible mechanisms of its action.

## Introduction

Our ability to successfully treat midlife diseases has increased human longevity as well as the prevalence of age-associated neurodegenerative diseases such as the Alzheimer's disease (AD). While a small percentage of AD is precipitated by dominant mutations, the majority is sporadic and is highly associated with environmental/life-style choices as well as the physiological aging process. In fact, aging is the strongest risk factor for development for AD, the most common form of dementia (1–3). As baby boomers age, millions of people are at risk for AD and other cognitive diseases. This is especially relevant as the number of AD diagnosis is expected to rise from current 5.7 million to 14 million by 2050, and the cost of disease is projected to rise to 1.1 trillion dollars creating a broad economic and social impact. Therefore, identifying therapeutic targets to delay, modify, or cure the disease is of paramount importance.

Menopause, a hallmark of aging in women, is associated with dysfunctions in the hypothalamic-pituitary-gonadal (HPG) axis and is implicated in the pathogenesis of AD. Age-related declines in reproductive hormones lead to a disarray of the HPG axis as it is tightly regulated by positive and negative feedback loops involving gonadotropin releasing hormone (GnRH), gonadal steroids, and gonadotropins. The loss of any component leads to significant dysregulation of the system and any type of hormonal imbalance likely affects homeostasis resulting in functional decline throughout the body, including the brain (4–8). In fact, these declines in gonadal hormones, and associated increases in gonadotropins such as LH are implicated in cognitive dysfunction in aging as well as the pathogenesis of age-related disorders such as AD. This is particularly relevant for women as they have two-fold higher risk for development of AD after menopause compared to men.

## Hormone replacement therapy (HRT) for cognition and AD

Several studies attribute increased risk of AD to the menopausal loss of estrogen (9, 10) and considered estrogen replacement a promising therapeutic avenue to reduce the risk of AD (11–14). This was initially supported by early epidemiological studies identifying strong relationships between estrogen therapy in post-menopausal women and reductions in multiple types of memory decline, specifically, in menopausal women with and without AD (15, 16).

Clinically, estrogen replacement or HRT showed initial promise in smaller clinical retrospective (17–20) and prospective studies (21–23). However, a large scale, randomized, double-blind placebo-controlled trial, women's health initiative WIH found no overall cognitive benefits. While the use of estrogen immediately or after a smaller delay following onset of menopause was effective, HRT in women at least 15 years after onset of menopause slightly increased the risk of dementia instead (24–26). This discrepancy in actions of estrogen on memory and dementia risk lead several groups to hypothesize that a “critical period” may exist following onset of menopause where estrogen can confer benefits, beyond which it is ineffective and may instead exert harmful effects (27–31). These clinical observations were further supported by pre-clinical work in ovariectomized rodents (32) and primates (33) providing support for the existence of a critical window for the initiation of estrogen for optimal enhancement of cognitive function (34).

One mechanism purported to underlie the ineffectiveness of estrogen treatment beyond a critical period is the “*healthy cell bias”* wherein estrogen is beneficial to healthy cells, but detrimental to unhealthy ones (35, 36). This was further supported by the fact that critical plasticity and cognition related proteins such as BDNF (37) and the cholinergic system (38) differ in young and old rats. Based on the above theory, more recent clinical randomized trials addressed the potential benefit of estrogen therapy considering timing of replacement. Unfortunately, these studies have yielded little support for this hypothesis (39–42). Together, these data suggest that while estrogen is relevant and important for CNS function and structure during the reproductive periods, aspects beyond estrogen loss contribute to age-related menopausal dysfunction.

## Involvement of luteinizing hormone (LH) in CNS function and AD

Accumulating evidence supports a role for LH in regulating cognition and AD-related parameters. LH, a gonadotropin hormone released from the anterior pituitary, functions to stimulate the production of sex steroids, which in turn negatively regulate hypothalamic GnRH release and further production of LH. In the absence of sex steroids providing negative feedback, LH levels drastically increase, as observed following menopause and andropause. Peripheral levels of LH increase three-fold in post-menopausal women (43) and two-fold in aging men (44). Importantly, these increases in peripheral LH levels correlate to cognitive deficits in aging men and women (45, 46) as well as AD patients (47–51). In fact, increased levels of peripheral LH in AD patients is associated with exacerbated pathology and cognitive deficits (50–52). Additionally, increased LH is correlated with increased plasma Aβ_1−40_ and Aβ_1−42_ levels in subjective memory complainers, suggesting LH may play a progressive role in early preclinical stages (51).

In rodents, earlier studies demonstrated that over-expression of LH in transgenic mice (53), or exogenous treatment with human LH (54) without any estrogen manipulations, impaired working memory. These studies are supported by work showing that chronic elevations in peripheral levels through exogenous application of the LH analog, human chorionic gonadotropin (hCG), results in similar attenuation of working memory as well as increases in total brain amyloid-β_1−40_ in a mouse model of AD (55, 56). Moreover, peripheral increase in hCG is capable of reversing estrogen associated benefits in spatial memory (55, 56).

Several studies involving pharmacological downregulation of peripheral LH after ovariectomy also support a role for LH in cognition and plasticity. To this end, work utilizing either GnRH super-agonist (57–60) or competitive antagonists (61–64) of the GnRH receptor reverse cognitive deficits (57–59–64) and neuronal plasticity loss (59, 60) in the absence of estrogen replacement. For example, several studies from our lab using GnRH super-agonist, leuprolide acetate, improved function in ovariectomized C57BL/6J mice (58, 60) as well as aged-ovariectomized 3xTg AD mice (59). Furthermore, these benefits were associated with activation of signaling cascades associated with long term potentiation (LTP), the cellular basis of learning and memory, Bryan et al. (58) in the absence of AD pathology (59), further supporting a direct role of LH-related mechanisms on cognition. More importantly, a key finding was that pharmacological reductions in peripheral levels of LH lead to cognitive improvements and rescue of dendritic spine density without any dependence on timing of treatment onset (60). This raises the exciting possibility that LH may provide an additional therapeutic target for rescuing cognitive and structural deficiencies associated with menopause, aging, and AD.

While there is no clinical data to support the benefits of peripheral downregulation of LH in menopausal women, clinical data in AD patients support its validity (65). A small, phase II, randomized, clinical trial, in female patients with moderate AD, showed that leuprolide acetate stabilized cognitive function in a subgroup treated with an acetylcholinesterase inhibitor, Aricept. Given Aricept is the primary treatment option for AD patients, these data are encouraging. However, while clinical and pre-clinical studies show promise for LH-related therapeutics, the mechanisms underlying these effects remain unknown to date. In the next sections, we highlight a potential mechanism of action of these therapies in relation to signaling and function of the LH receptor.

## LH signaling and cognition

LH as well its receptor LHR are expressed in the CNS (66–69). Importantly, LHR is present in regions associated with learning and memory (68–70). Similarly, LHR is also expressed in the choroid plexus, ependymal cells of the third, fourth and lateral ventricles as well as the area postrema (68), which are areas involved in production and circulation of cerebrospinal fluid CSF as well as blood brain barrier transport, suggesting a potential modulatory role of LHR in BBB permeability.

The LHR is a G-protein-coupled receptor involved in production of sex steroids in the gonads through Gs cAMP/PKA, ERK and Gq PLC pathways. G_s_ signaling results in phosphorylation of MAPK and CREB, while G_q_ induces intracellular Ca^2+^ release and activation of several protein kinases such as PKA and CAMKII (71–74). LHR activated cAMP/PKA signaling cascades in the immortalized cultures of hippocampal and hypothalamic neurons *in vitro* (75, 76). Interestingly, these signaling cascades downstream of LHR are associated with gene expression and structural changes that are the hallmark of synaptic plasticity and memory formation in the CNS (77–80) and induction of LTP (81–84). Furthermore, active kinases trigger activation of transcriptional factors such as MAPK and CREB leading to gene expression and structural remodeling of the synapse. This ultimately leads to enhanced synaptic transmission and BDNF production, as well as plasticity of dendritic spines (79, 85–87). However, as previously discussed above, downregulation of peripheral LH in the OVX model lead to CAMKII autophosphorylation at Thr 286 and CAMKII dependent GluR1 Ser 831 phosphorylation (58), inhibition of GSK3β and upregulation of β-catenin in 3X Tg mouse model of AD (59), and improved cognitive function. Based on the known canonical signaling cascades ascribed to the LHR, these data, at least indirectly, suggest activation rather than inhibition of the LHR, thus, in conflict with the prevalent idea that LH signaling in the CNS is detrimental.

The idea that LH signaling in the CNS is detrimental stems from the supposition that LH crosses the blood-brain-barrier (BBB); however, LH is a large glycoprotein that is unlikely to penetrate the BBB without a transporter molecule. Furthermore, both human and animal studies show contradictory evidence on whether hCG or LH can cross the BBB. One study showed some permeability of hCG into CNS when administered at supra-physiological levels (88) while another did not (89). Moreover, studies show hCG is not BBB permeable in human choriocarcinomas models until a high threshold is reached (90, 91), especially in patients with normal levels of plasma LH (92). These data suggest that hCG and LH may not be permeable in normal or OVX conditions, thus, the supposition that the LHR is detrimental is difficult to reconcile.

## Brain-specific LH as mechanism for LHR-mediated CNS function

Several earlier studies have demonstrated the expression of LH in the brain of various species (59, 66, 67), as well as its ability to modulate synaptic function (93–95), neurogenesis (96), and behavior (59, 88, 97, 98). *In vitro*, administration of the LH analog hCG, which binds and activates the LHR similarly to LH (99, 100), stimulates neurite outgrowth in rat neuronal culture and differentiation in PC12 cells through activation of the erk/MAPK pathway (99, 101). Erk/MAPK is a pathway that is critical for memory function and plasticity (78). Similarly, LHR activation *in vitro* also leads to stem cell differentiation (102), and protection against excitotoxicity in primary neurons (103).

LHR transcripts as well as functional receptors have been found in neurons and glial cells *in vitro* (94, 95). LHR was functional *in vitro* in neurons cultured at embryonic day 19. Similarly, hCG treatment of mixed glial cultures, from either P0 or P1 neonates, for 3 days lead to a dose-dependent increase in anti-inflammatory prostaglandin (PG), PGD2 and decrease in proinflammatory PGE2. These actions of LHR may be important for neonatal brain development and function as PGE2 inhibits the proliferation of glial cells, while PGD2 may do the exact opposite. Therefore, LHR signaling may be promoting controlled proliferation of glial cells during brain development (94).

While the above cited works cannot conclusively identify the source of LH in the brain, these studies demonstrate the presence of LH in cognition associated areas and its ability to mediate signaling and behavior. Furthermore, the highlighted *in vitro* work validates CNS LHR functionality and signaling consistent with cognition and neuroplasticity, both through neurons and glial cells. This said, how the LHR is involved in menopause and ovariectomy-induced cognitive and neuroplasticity loss remains unclear but may be explained through alterations in brain and peripheral levels of LH, as highlighted in the section below.

## Inverse relationship between periphery and the brain

Accumulating research shows that LH levels in the brain may have an inverse relationship to the periphery. For example, in studies of cycling adult female rats, hypothalamic LH decreased during proestrus, when a drastic 10-fold increase in LH occurs in the pituitary and periphery (104). Similarly, LH treatment into the median eminence (ME) of the hypothalamus in intact or castrated males and females significantly decreased both LH levels in the pituitary as well as the periphery (105). In a similar experiment, hCG treatment into the median eminence of hypothalamus in ovariectomized as well as intact mature female rats decreased peripheral LH (106). Additionally, LH mRNA levels are reduced in both the hippocampus and the cortex of human female AD brains (59), although they are increased in the periphery (47, 48). Interestingly, LH immunoreactivity is remarkably reversed when peripheral LH levels are downregulated and lead to improved cognition (59). These experiments, therefore, provide ample support to the idea of inverse relationship between peripheral LH and central LH, and further suggest that the LH and hCG in the periphery are capable of self-regulating their levels through short-loop feedback into the brain (105, 107, 108).

Interestingly, ovariectomized rats have no changes in hypothalamic LH, although the pituitary and serum LH levels are elevated eight-fold (104). Furthermore, the self-regulated decrease in peripheral LH in response to IV treatment of hCG and hLH seen in rabbits 2 weeks following castrations was absent 6 weeks following castrations, suggesting the sensitivity of the short-loop changes with time after castration (107). This begs the question of whether self-regulation of hypothalamic LH production is more pertinent than gonadal loss, and importantly, whether loss of this “short” feedback in menopause underlies the “critical period” sensitivity observed for estrogen. The fact that the LHR is rapidly internalized in the presence of its ligands (109–113) may provide a potential explanation for the loss of short-feedback loop, as impairments in LHR signaling due to ligand-induced internalization may reduce the ability to signal back to the hypothalamus. This may also explain the work postulating that activation of LHR is deleterious to cognition (52, 62, 88, 114) as the above studies used supraphysiological doses of exogenous hCG. In one of the studies above, deglycosylated hCG used as the LHR antagonist improved cognition; however, validation of the levels of LHR or verification of full deglycosylation of hCG was not performed (114). Therefore, further studies need to be carried out with careful consideration of LH dose and LHR internalization to clarify the role of LHR in the CNS.

An additional aspect that has confounded our ability to clearly dissect the function of the LHR in the CNS from that of loss of gonadal steroids is the fact that LHR has not been modulated in a brain-specific manner. For example, one article demonstrated that knocking out LHR in an AD mouse model attenuated the pathology and cognitive deficits (115). However, the LHR knockout model was global, not brain-specific, thus early developmental effects due to deficits in sex steroids, which are known in these animals, cannot be separated from the role of LHR loss.

## Conclusions

**Figure 1:**
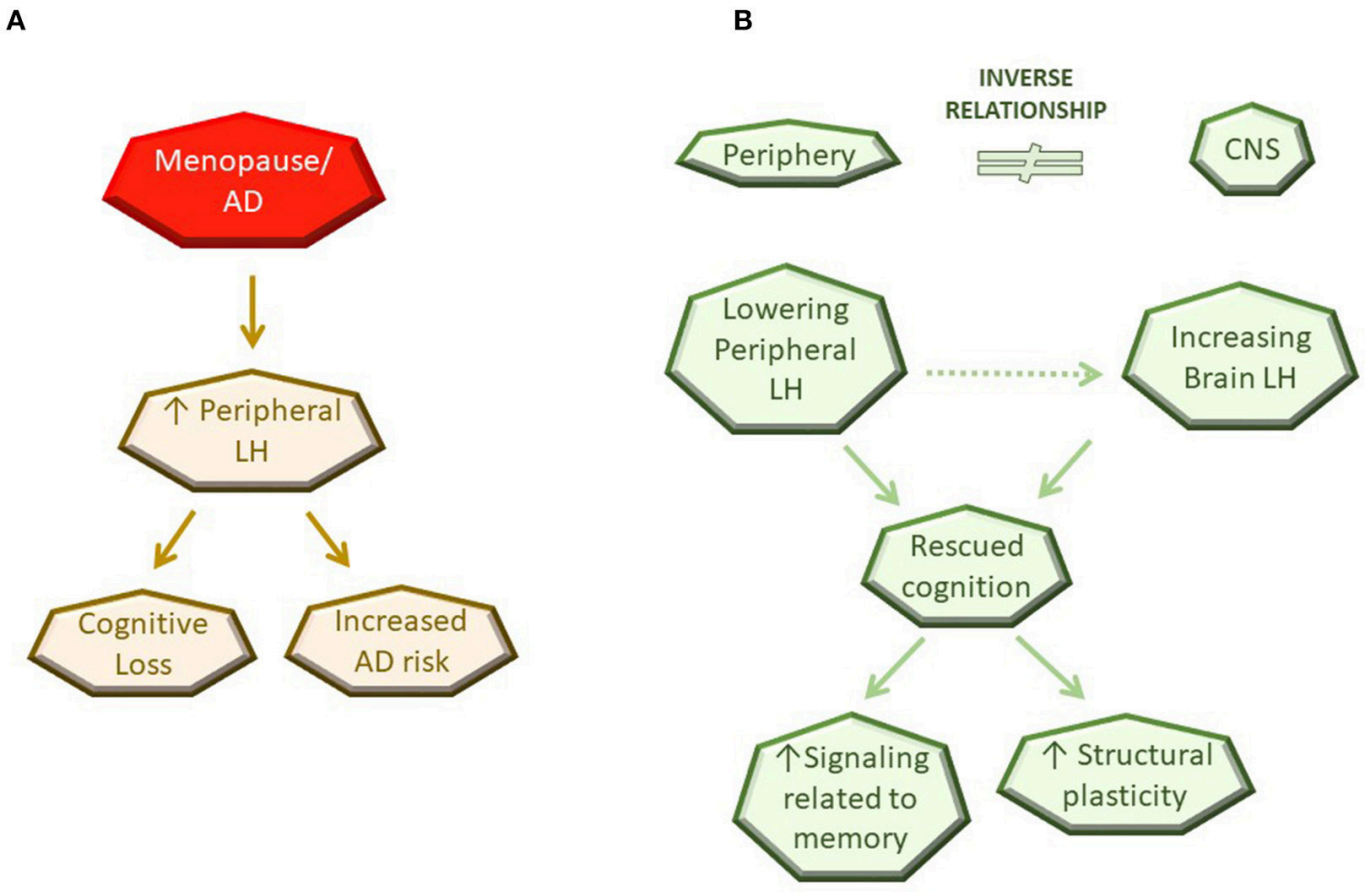
Schematic diagram of current working model for LH action in the CNS. **(A)** Observation in the literature. **(B)** Potential mechanisms of LH action.

Among a multitude of changes that occur with aging, menopause is a clear driver for development of AD in women. The role of steroid hormones in CNS function and structure, particularly during reproductive stages, is undisputed and here we provide evidence that LH and its receptor are important in CNS function as well as dysfunction as depicted in the working model of LH action in Figure 1. We also highlight a potential mechanism, specifically the inverse relationship of LH levels in the CNS vs. the periphery, to provide an explanation for the paradox observed between the ability of the LHR to activate cascades associated with learning, memory and neuroplasticity, and the findings demonstrating benefits of peripheral LH downregulation on these aspects. Further investigation is necessary to understand how the inverse relationship of LH between the periphery and the CNS is maintained as well as to identify the molecular mechanisms underlying LH-related memory and plasticity benefits in a CNS-specific manner. Genetic approaches targeting the LHR specifically within the brain are likely to be useful models to conclusively address the role of the LHR in CNS function and structure.

## Author contributions

All authors listed have made a substantial, direct, and intellectual contribution to the work and approved it for publication.

### Conflict of interest statement

The authors declare that the research was conducted in the absence of any commercial or financial relationships that could be construed as a potential conflict of interest.
